# The Work/Care Interface and Parents’ Mid-Pandemic Mental Health: Inequalities at the Intersection of Gender and High-Risk Household Status

**DOI:** 10.1177/21568693231223549

**Published:** 2024-02-14

**Authors:** Sylvia Fuller, Manlin Cai, Donna Lero

**Affiliations:** 1 The University of British Columbia, Vancouver, BC, Canada; 2 University of Guelph, Guelph, ON, Canada

**Keywords:** work-family interface, COVID-19, caregiving, gender, mental health

## Abstract

The COVID-19 pandemic generated mental health stressors for parents as they faced new health risks and navigated disruptions to employment, schooling, and care arrangements. Drawing on 2021 survey data from Canadian parents of children 10 years old and younger, we describe the relationship between work/care pandemic stressors and mental health, and employ Kitagawa-Oaxaca-Blinder decompositions to examine how these contribute to mental health gaps by gender and its intersection with having household members perceived to be at high risk in relation to COVID-19. We find that mothers’ mental health was more negatively affected than fathers’. Differences in exposure to work/care stressors help explain this gap, with the “mental load,” perceptions of inequity in how households responded to pandemic care demands, and greater reported deterioration in work-family balance and career prospects particularly salient. Mothers, but not fathers, with high-risk household members were also more exposed to key work/care stressors, contributing to the worst pandemic mental health for this group. While the relationship between stressors and mental health was similar for mothers and fathers overall, high-risk status moderated this relationship, with employment or care disruptions that reduced COVID-19 exposure less likely to be associated with poorer mental health for parents in high-risk families.

The COVID-19 pandemic has been difficult for parents. In addition to new health risks, parents juggled changing employment conditions and disruptions to schooling and care arrangements. The first wave of the pandemic in Spring 2020 was most difficult, with widespread job losses, the closure of schools and childcare centers, and guidelines around limiting contacts that reduced parents’ ability to call on relatives and friends for assistance with caregiving. Not surprisingly, multiple studies across countries found declines in mental health in the early months among parents, especially mothers, who experienced greater initial employment losses and bore the brunt of increased care demands (e.g., Gadermann et al. 2021; Montazer et al. 2022; Racine et al. 2021; Vicari, Zoch, and Bächmann 2022).

As the pandemic stretched on, new vaccines reduced COVID-related health risks for many. Yet vaccine rollout was much slower for children, and COVID-19 did not disappear. Although many aspects of work and family life resumed, they did not immediately return to prepandemic baselines. Gendered mental health impacts also lingered. For example, by late June 2020, American mothers (but not fathers) with school-aged children still had a higher probability of psychological distress (Zamarro and Prados 2021), and German mothers continued to have higher distress than fathers in March 2021 (Li et al. 2022).

Persistent mental health declines are especially concerning as chronic stressors can have long-lasting, cumulative effects on health and well-being (e.g., Pearlin et al. 2005; Qian and Fan 2023). Yet scholarship on parental mental health in later stages of the pandemic is limited. To the extent that disparities in mental health are examined, the approach has largely been to test the impact of differences in exposure to stressors—potential variation in their impact for mothers and fathers has received little attention (e.g., Cheng et al. 2021; Vicari et al. 2022; Zamarro and Prados 2021; but see Petts and Carlson 2023). Research has also tended to focus more on psychological resources than social determinants of mental health related to the ongoing reorganization of work and family life and associated supports (e.g., Louie, Upenieks, and Hill 2023; Montazer et al. 2022).

Moreover, although work-family scholarship has focused a great deal on the disproportionate burdens the pandemic posed for mothers (e.g., Fuller and Qian 2021; Haney and Barber 2022; Waddell et al. 2021), there has been surprisingly little consideration of how household health risks affected challenges at the work/care interface. Harms associated with the pandemic have been unevenly distributed, with more marginalized groups often bearing the brunt (Brown and Ciciurkaite 2022; Pettinicchio et al. 2021; Qin et al. 2022). Health status, such as being immunocompromised, emerges as a particularly salient marker for mental health during a viral pandemic (Islam et al. 2021; Smith et al. 2021). A COVID-19 infection can result in serious short- and long-term outcomes for anyone, but health risks are not equal. Greater vulnerability not only affects individuals but also poses work/care challenges to other household members and functions as a family-level stressor. The pandemic experiences of parents in high-health-risk families differed from others, particularly as governments, schools, and employers rolled back protective measures and pandemic responses became increasingly politicized. Without societal protections, such families were left to develop their own work/care strategies as best they could. This could increase demands for parental caretaking and family coordination, threaten relationships, and spread stress to all family members, magnifying threats to mental health and potentially creating differences in how particular stressors were experienced. Given the salience of gender at the work/care interface, family health-risk status may also have had different consequences for mothers and fathers’ mental health, making it important to consider these social statuses in tandem.

In this article, we draw on survey data collected a year and a half into the pandemic from Canadian parents of children 10 years old and younger to explore the relationship between changes at the work/care interface and gender gaps in mental health. We further analyze within-gender differences among those who do and do not report having a household member at high COVID-19 risk. In so doing, we extend research documenting gendered mental health gaps among parents early in the pandemic by investigating the extent to which these persisted in later stages characterized by less universal disruption to work and care arrangements, considering a broader array of work/care covariates than much previous research, including stressors of particular relevance to later pandemic stages, and revealing key divergences in the gendered pandemic work/care stress process for families with members at high risk of COVID-19.

## Stress and Mental Health

Pearlin’s (1989) stress process paradigm had been foundational to the sociological study of mental health and serves as a guiding framework for our investigation. First articulated over 30 years ago and taken up broadly and elaborated since (Turner 2010), the model highlights how stress exposure can arise from both discrete life events and chronic strains tied to social contexts, and how psychological and social resources shape how people appraise stressors, and the degree to which they affect mental health. Rather than focus on single stressors in isolation, the stress process model emphasizes how stress accumulates as stressors combine, and how stressors at one point in time can proliferate to have downstream effects through creating new or secondary stressors, and spill over between life domains and across people whose lives are linked (Pearlin 1989; Pearlin et al. 1981). Given that family members lead interdependent lives, family-level dynamics comprise a vital component of the stress process (Milkie 2010; Young, Schieman, and Milkie 2014).

For parents, the pandemic created multiple stressors at the work/care interface (Montazer et al. 2022). Workplaces, schools, and childcare centers became sites of infection that could then be transmitted within households. Public health orders affecting these domains generated secondary stressors via increased demands for parental caretaking, which can heighten work-family conflict (Bianchi and Milkie 2010; Montazer et al. 2022). Navigating pandemic risks, employment, and care demands could also necessitate role restructuring around earning and caregiving—also a potential stressor (Milkie 2020; Pearlin 1989). Meanwhile, growing politicization of pandemic responses exacerbated differences in the appraisal of COVID-19 risks and magnified mental and emotional aspects of care work critical to maintaining family relationships and well-being (Calarco et al. 2020; Petts and Carlson 2023).

While such circumstances potentially expose all parents to greater levels of pandemic stress than their childless counterparts (Montazer et al. 2022), we expect gender and family health-risk status to moderate these dynamics. According to the stress process paradigm, mental health gaps may emerge as life circumstances tied to salient social statuses shape both exposure to potential stressors and access to material and psychosocial resources that can buffer stressors’ effects (Pearlin 1989). People thus vary in their vulnerability to stressors. Extant scholarship has demonstrated the salience of gender in pandemic inequalities concerning work/care, particularly among parents (e.g., Fuller and Qian 2021; Haney and Barber 2022; Montazer et al. 2022). For example, given patterns of occupational segregation coupled with gendered caregiving norms, mothers were more likely to lose their jobs and have their employment hours reduced than fathers early in the pandemic, and they experienced a slower employment recovery (Fuller and Qian 2021; Qian and Fuller 2020). Although fathers initially responded to increased needs by ramping up their involvement in caregiving and housework, mothers nonetheless shouldered more pandemic caregiving labor (Carlson, Petts, and Pepin 2020; Fuller and Qian 2021; Shafer, Scheibling, and Milkie 2020).

Family health status is likewise an important social status in pandemic times. Groups marginalized by ableism are typically exposed to greater stress, face unique stressors, and have fewer resources to buffer stress exposure (Brown and Ciciurkaite 2022; Meyer 2003; Meyer and Frost 2013; Montazer et al. 2022). For those with family members at high-health-risk (henceforth “high-risk” families) in the pandemic, managing work and care has required a higher level of adaptation (Driessens et al. 2023). Gendered dynamics may also be exacerbated, with mothers feeling extra pressure to protect vulnerable family members.

In what follows, we delve deeper into the specifics of how differences in both exposure and vulnerability to stressors could affect mental health gaps at the intersection of gender and high-risk family status. The proliferation and complex interplay of stressors mean that they often operate in clusters, and that different stressors may have offsetting effects across groups. A narrow focus can thus mislead as to overall differences in stress exposure (Pearlin 1989; Schieman 2010; Wheaton 2010). This is apparent with respect to pandemic stressors, especially those operating at the work/care nexus, highlighting the importance of a broad assessment of multiple stressors (Louie et al. 2023). We therefore consider in tandem multiple dynamics related to care demands, employment, and their intersection.

## Pandemic Parental Mental Health and the Work/Care Interface

### Care Demands

When the pandemic first hit, schools and childcare centers across Canada were typically closed. Schools then moved online, with only Quebec fully reopening in-person schooling before the school year’s close (Childcare Resource and Research Unit 2020; Han and Breton 2022). Parents thus lost access to resources critical to meeting children’s care and educational needs. All provinces returned to in-person schooling at the start of the 2020–2021 school year, and regulated childcare centers had almost all reopened by late August 2020, but subsequent waves resulted in some province-wide closures for varying periods as well as regional closures (Han and Breton 2022; Vickerson et al. 2022). Attending to children required to isolate after COVID-19 exposures also increased caregiving demands, albeit for shorter periods. Past experiences with disruptions may have produced anticipatory stress, given the pandemic’s uncertain trajectory.

Although there is no reason to expect differences in exposure to closures, moves online, or isolation requirements by gender or high-risk status, vulnerability to these stressors may vary. Fathers spent more time caring for children early in the pandemic (Shafer et al. 2020), but heightened care demands were still disproportionately borne by mothers, contributing to gender gaps in well-being (Etheridge and Spantig 2022; Pierce et al. 2020; but see Adams-Prassl et al. 2020). This suggests past exposure to care disruptions will create higher anticipatory stress for mothers, widening mental health gaps.

Differences in how people appraise the meaning of stressors can also moderate their impact (Milkie 2020). Having to isolate children who contracted or were exposed to COVID-19 reinforces parental awareness that daily routines risk viral exposure. This could generate stress for all families, especially as vaccines were not available for children at the time of our survey. However, we expect heighted awareness of transmission risks would be more threatening to the mental health of parents in high-risk families, widening the mental health gap between them and others. School and childcare closures and remote schooling, on the other hand, sheltered children from infection. This should lead to differing appraisals of their meaning for parents in high-risk families. While losing access to care and schooling was no less challenging, associated stressors may have been offset by reduction in health risks, narrowing mental health gaps.

Like closures and isolation requirements, withdrawing children from childcare or in-person schooling exacerbated caregiving demands, a stressor for mothers especially. However, problem-focused coping, where actions reduce sources of stress, can be adaptive and positive for mental health (Lazarus and Folkman 1984). Being able to limit family exposure to pandemic health risks could thus be a coping strategy. Given greater vulnerability to serious pandemic health risks, we expect withdrawal to be more common for high-risk families, which could offset mental health gaps. The strength of stress-buffering from this resource may also vary. Keeping children home reduces threat, and hence stress, more profoundly for high-risk families, especially for fathers as they were less likely to bear the brunt of increased care demands.

Whether resulting from closures, isolation, or parental choice, one’s role in meeting caregiving demands matters. Canadian fathers are more likely than mothers to perceive that they spend too little time with their children, which is associated with psychological distress (Milkie, Nomaguchi, and Schieman 2019). Therefore, increases in caregiving time could be more positive for fathers’ mental health than for mothers’. For high-risk families, the greater riskiness of calling on friends or family reduces access to care resources, increasing the likelihood that increased time with children will be experienced as a stressor.

Feeling that burdens are shared can be a source of social support, whereas feeling that burdens are borne alone may further stress (Claffey and Mickelson 2009; Waddell et al. 2021). Despite fathers’ increased involvement in caring for children early in the pandemic, mothers still reported taking on a larger share of pandemic caregiving than their spouses (Haney and Barber 2022; Shafer et al. 2020). Greater exposure to this stressor would widen the gender mental health gap. This stressor may also be more threatening for mothers’ mental health insofar as it exacerbates extant gender inequalities, prompting concerns about unfairness. Inequality in time spent caring for children is not necessarily seen as inequitable given factors such as partners’ other time commitments, subjective perceptions of entitlement, competence, preferences, and comparison referents outside the couple relationship (Thompson 1991; Young, Wallace, and Polachek 2015). Nonetheless, increases in women’s relative childcare contributions typically magnify perceptions of unfairness, especially among women (Koster et al. 2022). Notably, when Canadian fathers were more involved in pandemic childcare, this increased their women partners’ (but not their own) satisfaction with the division of labor (Petts et al. 2023). However, role reorganization can also be stressful (Pearlin 1989; Thoits 2013). Fathers’ taking on the lion’s share of increased demands for care may threaten established gender roles, provoking greater stress and narrowing the gender mental health gap.

While challenges meeting children’s increased needs for direct care and supervision may be the most obvious pandemic parenting stressors, the pandemic also increased demands for cognitive, emotional, and relational elements of care work. Recognizing needs, thinking about solutions to meet them, monitoring outcomes, and caring for others’ emotions are important aspects of care work that together contribute to parents’“mental load” (Dean, Churchill, and Ruppanner 2022; Doucet 2023; McKeown 2021; Robertson et al. 2019). Managing health risks while meeting family members’ needs for sociability, support, and connection with friends and family outside the household required parents to think about and recognize an array of potentially competing needs within families, consider the risks and benefits of different ways of meeting them, monitor family members for symptoms of COVID-19, and manage the emotional fallout of often difficult choices that could disappoint children and other family members. This took place, moreover, alongside growing differences in perceptions of pandemic risks and appropriate mitigation strategies (Grossman et al. 2020; Qian, Xie, and Jin 2022; Wu and Huber 2021). This made coordinating with others within and outside households to meet needs increasingly contentious (Calarco et al. 2020). Challenges in maintaining relationships, getting along, and managing children’s behavior can be a potent source of stress.

The mental load is highly gendered as mothers take the lion’s share of recognizing family needs, thinking about and monitoring the solutions (Daminger 2019), and caring for family members’ emotions (Hochschild 2003). Even when mothers and fathers spent similar time considering family issues, family-related concerns were associated with lower emotional well-being only among mothers (Offer 2014). Mothers not only carry more of the load but may also feel the weight more. We therefore expect mothers to perceive greater responsibility for and concern about family functioning in the pandemic and be more vulnerable to the negative effects of mental load, with both contributing to the gender mental health gap.

The mental load related to family functioning is also likely to be especially heavy for high-risk families. As others abandoned precautions, maintaining connections outside the household became more fraught for high-risk families. Keeping safe increasingly meant missing aspects of social life available to others and visibly standing out as different in ways that invited stigma (Kassa and Pavlopoulou 2021; Seiter, Curran, and Elwood 2023). Decisions about how best to safeguard families’ emotional and physical well-being became more difficult as the pandemic wore on and the toll of being left behind deepened (Giles 2022). Moreover, the abandonment of efforts to prevent infection and transmission could leave high-risk families questioning whether they and their loved ones mattered to others (Yong 2022; Zomerdijk et al. 2022). Mattering, a belief in one’s importance to other people, is important for mental health (Rosenberg and McCullough 1981; Schieman and Taylor 2001; Thoits 2011). Its erosion could increase the mental load associated with managing relationships and meeting family members’ emotional needs, thus widening the mental health gap by family risk status.

### Employment

Job loss and unemployment are serious life stressors (McKee-Ryan et al. 2005; Paul and Moser 2009; Pearlin et al. 1981). The stigmatizing aspects of pandemic job loss should have been reduced by its widespread nature and clearly external locus, but it can still strain finances and reduce access to important sources of social connection and support, as can other employment disruptions that spiked early in the pandemic, such as furlough, leave-taking, and reduced working hours.^1^ Not surprisingly, research has found a negative association between pandemic job loss and mental health (Borrescio-Higa and Valenzuela 2021; Etheridge and Spantig 2022; Racine et al. 2021). While employment had rebounded by the time of our survey (Clarke and Fields 2022), we expect that having experienced such disruptions could still prompt anticipatory stress, given the pandemic’s uncertain course.

Gendered patterns of occupational segregation and caregiving affected parents’ exposure to the stress of pandemic employment disruptions, with mothers more affected than fathers (Fuller and Qian 2021; Qian and Fuller 2020). We expect differences in exposure to such disruptions to widen gender mental health gaps among parents. Although there is a dearth of research on this topic, it is reasonable to expect that parents in high-risk families would also be more exposed to these stressors if the more threatening nature of viral exposure pushed them out of in-person jobs or motivated leave-taking or hour reductions to meet greater care demands.

High-risk families may have also been affected differently. As in-person work magnified risk of contracting COVID-19, especially after protective measures were revoked, members of high-risk families who lost their jobs likely faced greater difficulty job-hunting, given concerns with employment-related viral exposure. However, as with care disruptions, changes that reduced viral exposure without entirely severing employment relationships, such as furlough, working remotely, and working hour reductions, could buffer health-related stress.

Employment disruptions are not only relevant for one’s current situation, they can also affect future employment trajectories (Fuller 2008; Pedulla 2016), proliferating stress via concerns about future career prospects. Pandemic uncertainties about the ongoing availability and safety of schooling and childcare, and of workplace safety measures and accommodations for caregiving, could also create stress about career prospects even for parents who did not experience disruptions. Given mothers’ greater role in meeting care needs and more negative consequences of viral transmission at work for high-risk families, we expect mothers and parents in high-risk families to experience heightened concerns about their career prospects.

### Balancing Work/School and Caring for Children

Work-family conflict causes strains across role domains (Nomaguchi and Milkie 2020). Not surprisingly, Racine et al. (2021) found that mothers who reported difficulties managing their own schooling/employment and obtaining childcare in May to July 2020 reported worse declines in mental health. Employment and care stressors and associated adaptations discussed above intersect and combine to shape exposure to work-family conflict. So too can additional pandemic work/care adaptations such as changes in children’s extracurricular activities or shifts to working from home that we cannot directly measure with our data. Given cultural norms that pressure mothers to prioritize family needs (Collins 2019; Mathieu et al. 2023; Young et al. 2014) and less flexibility of high-risk families to meet these needs due to the importance of avoiding infection, mothers and parents in high-risk families may experience higher conflicts between work and care demands, with the intersection of gender and high-risk status creating particularly strong threats for mothers in high-risk families.

As the preceding discussion illustrates, a year and a half into the pandemic, the work/care interface remained a potent potential source of numerous interlocking stressors threatening parents’ mental health. Table 1 shows a more formal summary of the predicted impacts of these stressors on mental health gaps among parents by gender and health status.

**Table 1.** Predicted Relationships between Stressors/Buffers and Mental Health Gaps.

Stressor/buffer	Greater exposure for	More negative relationship for	Relationship to gender mental health gap	Relationship to high-risk mental health gap
Care demands				
School/care closure				
		Mothers/not high risk	+	−
School/care isolation				
		Mothers/high risk	+	+
School/care withdrawal	High risk			−
		Mothers/not high risk	+	−
Change in time spent caregiving	Mothers/high risk		+	+
		Mothers/high risk/mothers*high risk	+	+
Change in share of caregiving	Mothers		+	
		Mothers (fairness); fathers (role restructuring)	±	
Change in mental load	Mothers/high risk		+	+
		Mothers	+	
				
Employment				
Job loss	Mothers/high risk		+	+
		High risk		+
Self-initiated reduction in hours	High risk			+
		Not high risk		−
Employer-initiated reduction in hours	Mothers		+	
		Not high risk		−
Furloughed	Mothers/high risk		+	+
		Not high risk		−
Concerns about declining career prospects	Mothers/high risk		+	+
Change in difficulty balancing work/ school and childcare	Mothers/high risk		+	+

*Note.*“+” signifies widening and “−” signifies narrowing/offsetting of the mental health gap disadvantaging mothers versus fathers or parents in high-risk families versus non-high-risk families.

## Data and Methods

Our data come from the Canadian component of the cross-national “familydemic” survey. The 30-minute survey was fielded between August 20 and September 6, 2021, and included 4,683 parents aged 20–55 years with coresident children 10 years old or younger. It employed a quota design linked to provincial population size, with minimums for single parents, Indigenous parents, the Lesbian, gay, bisexual, transgender, queer + (LGBTQ+) population, and across racial minority groups. The survey was fielded by the Angus Reid group, with respondents paid a modest sum for participation. Survey details can be found in Kurowska et al. (2023).

As we are interested in pandemic-related employment changes and mental health, we include men and women employed prior to the pandemic (*N* = 3,905). We then drop 397 individuals who were self-employed prepandemic as employment changes for them differed. We keep former employees who transitioned into self-employment during the pandemic as this may be an important adaptation. Given our interest in domestic divisions of labor, we restrict analysis to partnered parents, dropping a further 278 respondents. After listwise deleting 448 observations with missing values, our final sample includes 2,782 parents.

### Variables

Our analysis uses a global measure of mental health. Respondents were asked, “Would you say your mental health was/is excellent, very good, good, fair, poor? [before COVID-19/currently].” This measure is commonly used in health research and is correlated with multi-item measures of mental health (e.g., psychiatric disorders; for a review, see Ahmad et al. 2014). Answers referencing current mental health are the dependent variable, treated as continuous. Results were substantively similar when using ordinal logistic regression.

Our gender measure asked participants “What is your gender identity?” Responses were woman, man, nonbinary, other (with respondents invited to specify with a fill-in option), and “prefer not to say.” The question had a note: “We understand ‘woman’ and ‘man’ to be inclusive of both cis and trans identities.” Here, we include those who answered “woman” or “man” as the number of respondents who chose nonbinary was too small to be meaningfully analyzed.

To identify households with members particularly vulnerable to COVID-19, we asked, “Do any members of your household have a health condition that puts them at higher risk of poor outcomes from COVID-19 (yes/no)?.” Individuals may not fully understand the relationship between COVID-19 risk and health conditions of household members. Our inability to rely on more objective indicators of risk or to consider variability in its degree is a major limitation of our study. However, the National Advisory Committee on Immunization developed guidelines to identify individuals at higher risk from COVID-19. Canada’s universal health system provides unique personal health identifiers that were combined with health and prescription databases to identify those at highest risk, and these people were contacted with invitations to be vaccinated earlier than others. The nature of vaccine rollouts in Canada thus makes it highly likely that people were aware when household members were deemed to be at higher risk by health experts. Moreover, perception of health risks is itself socially salient and affects how people respond to the pandemic.

#### Care demands

We measure *disruptions to care supports* over the course of the pandemic. The survey asked how long preschool-aged children were not in their usual care arrangement and how long school-aged children were staying at home for various reasons: care/school closure and/or moving online, isolation/quarantine requirements, and parental decision to keep children home (no disruption/less than once a month/about a month/about 2–3 months/about 4–5 months/6 months or more). We refer to questions for both preschool- and school-aged children to capture the longest time school or care was disrupted for a child in the household for each reason. The distribution of responses to these questions varied. We considered the nature of distributions, differences between categories in their relationship with mental health, and model fit to determine the best operationalization of each variable. For disruptions due to closures, we combine all responses for closures of up to 4 months, differentiating these from those of 4–5 months, and 6 months or more, and treating this measure as interval. We collapse the quarantine measure into none, less than a month, and about a month or more, and the measure of keeping children home to less than a month, about a month, about 2–3 months, about 4–5 months, and 6 months or more. These two variables are treated as categorical.

We include two measures of pandemic *changes in parent’s time caring for children*. The first measures perceived change in overall time spent in caring for children and household work (much less time than before/somewhat less time than before/about the same amount of time as before/more time than before/much more time than before). The second captures relative change with a binary variable combining answers to questions about one’s own and one’s partner’s change (whether care and household workload increased more/decreased less than partner).

Pandemic-related increases in the *mental load* are operationalized via answers to concerns around family functioning with questions that asked, “Since the COVID-19 outbreak, how concerned have you been about the following for your family”: staying connected with family or friends; getting along and supporting each other; managing your child’s/children’s behaviors, stress, anxiety, emotions; having less patience, raising your voice, scolding or yelling at your child or children. Answer categories were much less concerned, somewhat less concerned, the same, more concerned, and much more concerned, and were converted to a numerical scale (1–5), summed, and averaged to create an index. Principal components factor analysis confirmed that each question highly loaded onto one factor (Cronbach’s alpha = .72).

#### Employment

In the employment domain, to capture pandemic *employment disruptions*, we measure whether the respondent experienced losing a job, lay-off/furlough, employer-precipitated reduction in working hours, self-precipitated reduction in working hours, and/or leaving one’s job for caregiving or health reasons since the COVID-19 outbreak. *Assessment of career prospects* is a 5-point scale derived from a question asking, “Comparing the current situation with the month before COVID-19, my career prospects: improved a lot, somewhat improved, did not change, somewhat deteriorated, deteriorated a lot.”

#### Balancing work/school and caring for children

Respondents were asked how concerned they are/were since the COVID-19 outbreak about “balancing childcare with your paid work and/or schoolwork” (much less concerned/somewhat less concerned/the same/more concerned/much more concerned). We treat this as a linear scale.

#### Controls

Group differences in current mental health may reflect variation in preexisting mental health issues. Women, people with chronic illness, and parents of children with serious health conditions were more likely to report poor mental health even before the pandemic (e.g., Banks and Xu 2020; Brehaut et al. 2009; Pettinicchio et al. 2021). To account for this, we include respondents’ assessment of prepandemic mental health. We control for the respondent’s current employment status measured by five categories: employee, self-employed, on leave, unemployed, and out of labor force.^2^ The co-occurrence of disability and chronic health conditions tied to higher COVID-19 risk is considerable, but not complete (Centers for Disease Control and Prevention 2022, 2023). Some disabled people are not at elevated risk, while others who may not have identified as disabled prepandemic are (e.g., people with diabetes or conditions controlled by immunosuppressant drugs). To isolate the impact of COVID-19 health risk, we control for disability and individual age. To account for family-level dynamics relevant to the stress process, our models also control for whether one’s child and one’s partner have a disability, age of the youngest child, household income, and partner’s employment status.

### Estimation Strategy

We use Kitagawa-Blinder-Oaxaca decomposition models (Kitagawa 1955; Oaxaca 1973; Oaxaca and Ransom 1994) to assess how covariates contribute to mental health gaps. These decompose group differences into three parts: differences in the mean of independent variables (endowments); differences in their effects (coefficients); and an interaction between mean and coefficient differences (residual). We examine group differences in mental health first by gender among all parents and then by high-risk household status among fathers and mothers separately.

## Results

**Figure 1.**
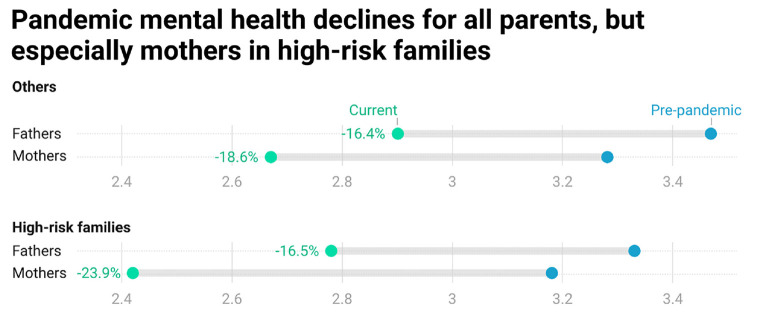
Unadjusted pandemic mental health scores by gender and high-risk family status. *Note.* Figures created with Datawrapper.

To provide a baseline for understanding group differences in mental health, Figure 1 presents unadjusted pandemic mental health scores by gender and high-health-risk family status. Other descriptive statistics can be found in Online Appendix A. Of note, all parents report lower mental health than prepandemic. Mothers not in high-risk families report both lower prepandemic mental health than fathers and significantly greater declines (−18.6 vs −16.4 percent). Fathers in high-risk families (23.4 percent of fathers in the sample) report lower prepandemic mental health than other fathers but declines of a similar magnitude (−16.5 percent). Mothers in high-risk families (27.5 percent of mothers), however, report both lower baselines and greater declines (−23.9 percent) than other mothers. Gender differences in pandemic mental health are thus magnified in high-risk families, with these mothers facing the greatest pandemic mental health burden of all groups.

### Work/Care Stressors and Gender Differences in Pandemic Mental Health

**Figure 2.**
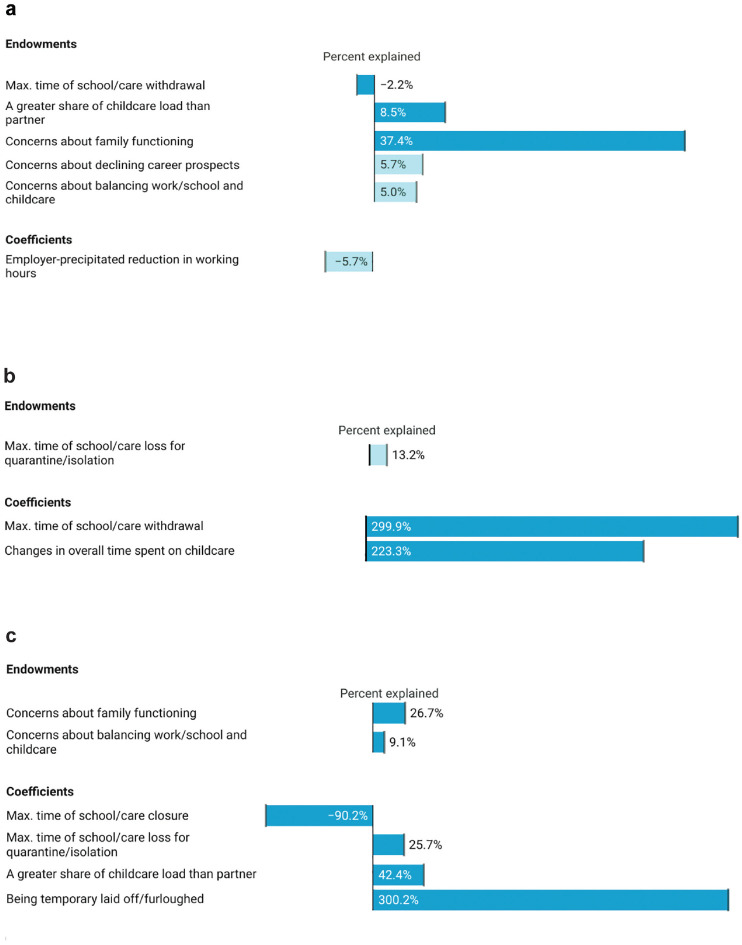
Mental health gaps explained by endowments and coefficients. (a) Gender gap. (b) Gap between high-risk families and others, fathers. (c) Gap between high-risk families and others, mothers. *Note.* figure created with Datawrapper

To explore how stressors of work, care, and their intersection relate to these pandemic mental health gaps, we first decompose the overall gender mental health gap. Figure 2a shows covariates that significantly contribute to (or offset) the gender mental health gap either through differences in men and women’s means (exposure to stressors) or their coefficients (differences in the relationship between stressors and mental health). Dark bars indicate covariates significant at *p* < .05, while light bars indicate *p* < .1. Full results of the decomposition are in Online Appendix B1. As choice of base category can affect decomposition results, we normalize all categorical variables so that effects are expressed as deviations from the grand mean. However, when discussing the general relationship between categorical covariates and mental health, we refer to estimates relative to a base category instead where this is more intuitive (e.g., comparing relative to those whose caregiving time did not change—detailed results available on request).

Overall, there is a 9.3-percent gender gap favoring men in current mental health. Although we thought that care disruptions would be more detrimental for mothers’ mental health, the relationship between past care disruptions and mental health does not differ by gender. In fact, none of the care disruption variables are significantly associated with current mental health net of other covariates except for withdrawal for women (a nonlinear pattern). A small difference in exposure to withdrawal offsets the mental health gap, but the impact is small (2.2-percent reduction).

We thought that fathers might benefit from additional time with children. In fact, spending both more and less time predicts worse mental health for fathers relative to those whose time did not change (it is neutral for mothers). Differences in mental health compared with fathers who did not change their caregiving time are largest and significant for fathers spending somewhat less and much more time with children than before the pandemic.

While changes in absolute time caring for children do not contribute to the overall gender mental health gap, perceived change in how caretaking is allocated between partners does. Mothers are more likely to report that they took on a greater share of pandemic childcare than their partner, with the stress associated with this type of role reorganization accounting for 8.5 percent of the gap. Coefficients for this variable do not differ significantly for mothers and fathers. We also find a similar relationship between concerns about family functioning and mental health for mothers and fathers. However, as expected, mothers are more exposed to this stressor, which accounts for a substantial 37.4 percent of the gender mental health gap.

None of the pandemic employment disruption variables independently predict current mental health. Mothers are, however, more likely to experience heightened concerns about their career prospects. This difference accounts for 5.7 percent of the gender mental health gap among parents.

Estimated without other work/care covariates, reporting greater concerns about balancing work/school and childcare since the pandemic has a negative association with mental health for both mothers and fathers. However, the effect of this covariate is much reduced and only significant for mothers in our full decomposition model, indicating that changes in parents’ work/care balance are largely captured by other measures of employment and care changes. Mothers are more likely to report heightened concerns about balancing work/school with childcare than are fathers; all else equal, this difference in exposure explains 5.0 percent of the gender gap in parental mental health.

Overall, our decomposition reveals that gender differences in the relationship between work/care covariates and mental health explain little of the gender gap. A year and a half into the pandemic, mothers and fathers experienced potential work/care stressors in similar ways. However, mothers were more likely to experience key work/care stressors that are correlated with worse mental health, with greater increases in the mental load related to concerns about family functioning contributing particularly strongly to the mental health gap.

### Differences in Pandemic Mental Health at the Intersection of Gender and High-risk Status

The next analyses consider the intersection of gender and high-risk status, analyzing mental health gaps between high- and low-risk families among fathers (Figure 2b, Online Appendix B2) and mothers (Figure 2c, Online Appendix B3).

#### Fathers

Among fathers, the mental health gap by high-risk family status is small at 2.5 percent and not significant. Contrary to our expectations, we fail to find greater exposure to caregiving demands or mental load or a more negative effect of children’s isolation time for fathers in high-risk families. However, some work/care covariates affect fathers in high-risk families differently. Fathers in high-risk families report keeping children home longer (*p* = .083), and the impacts of choosing to withdraw children also differ. For fathers who do not report having a household member at elevated risk, a brief withdrawal of less than a month is positively associated with mental health. Having some flexibility to pull one’s child from school for a brief period appears protective. For fathers in high-risk families, withdrawing one’s child is also protective, but a longer period is required. Both no withdrawal and withdrawal of less than a month are negatively associated with mental health relative to the grand mean. However, most fathers in high-risk families report that they did not withdraw their children or did so for less than a month. Thus, while differences in exposure to withdrawal from school/care offset the gap to a small degree, differences in coefficients grow it by 299.9 percent.

Looking more directly at caregiving time reveals further differences. Among fathers not in high-risk families, less time spent in childcare than before the pandemic predicts significantly worse mental health relative to those who did not experience change; increases are also negative but not significantly so. For fathers in high-risk families, spending somewhat less time is also negative (*p* = .080). However, spending more time (much more common for both sets of fathers than decreases) is also worse than no change. Consistent with our expectation that differential vulnerability to the stress of additional caregiving time would widen the mental health gap between high-risk families and others, and this coefficient difference grows the gap by 223.3 percent.

None of the employment variables significantly affect mental health differences among fathers. However, it is worth noting that leaving one’s job for care or health reasons is associated with better mental health for fathers in high-risk families but not others. There are no significant differences in either exposure or consequences with respect to the relative division of caregiving labor, the burden of mental load, or work-life balance among fathers.

The small and nonsignificant overall gap is thus not indicative of the total irrelevance of high-risk status for work, care, and mental health among fathers. Differences in relation to keeping children home from care/school and time caring for them contribute to predictions of worse mental health for fathers in high-risk families. These differences, however, are offset by some differing relationships for controls (notably, a more negative relationship between having a partner with a disability and mental health for fathers not in high-risk families).

#### Mothers

The mental health gap between high-risk and other families is significantly higher among mothers (9.1 percent) and is of similar magnitude to the overall gender mental health gap. Exposure to care disruptions does not differ by family health status, nor does the relationship between voluntary withdrawal of children from school/care and mental health. However, high-risk family status moderates the relationship between both school/care closures and isolation requirements and mothers’ mental health. The former have no significant relationship with mental health for mothers in high-risk families but a negative association for other mothers, reducing the mental health gap by a substantial 90.2 percent. However, differences in the latter exacerbate the gap by 26.7 percent. Children’s isolation is not significantly related to current mental health for mothers without high-risk family members, but, contrary to expectations, having to isolate one’s child for a longer period is positively related to mental health for mothers in high-risk families.

Although differences in time spent caring for children contribute to mental health differences by health risks among fathers, this is not the case for mothers. The distribution of caretaking between partners does matter. Perceiving that one took on a greater share of pandemic caregiving demands than their partner is associated with worse mental health for both sets of mothers, but the relationship is stronger and only significant for mothers in high-risk families. This differential effect grows the gap by 42.4 percent. Mothers in high-risk families also report greater concerns about family functioning, with this exposure difference increasing the gap by 26.7 percent.

Whereas employment variables are not explanatory among men, furlough has a strong negative relationship with mental health for mothers in high-risk families but is protective for other mothers, increasing the gap by 300.2 percent. While not contributing significantly to the mental health gap, it is also worth noting that reductions in work hours are more positive for mothers in high-risk families. Self-precipitated reductions in work hours are neutral for these families but negatively related to mental health for other mothers. Employer-precipitated reductions in working hours are positively related to mental health for mothers in high-risk families but neutral for other mothers. Mothers in high-risk families also report a greater deterioration in their ability to balance paid work/school and childcare, which explains 9.1 percent of the mental health gap.

## Discussion

For previously employed Canadian parents, the mental health impacts of the pandemic did not end after the first wave. A year and a half in, mothers and fathers still perceived their mental health to be worse than prepandemic baselines. Mothers were, however, harder hit. The gender mental health gap was not attributable to scarring effects of the dramatic disruptions to employment and care arrangements that were most evident in the initial “lockdown” period of the pandemic and highlighted in much prior work on parent’s pandemic mental health. Nor were gendered patterns in changes to absolute time spent caring for children the most important. Instead, it was perceptions of inequity in how households responded to increased care demands that mattered, along with mothers’ greater perception that, all else equal, the pandemic led to a deterioration in overall work/care balance and career prospects.

The importance of perceptions of the relative distribution of changes in time spent caring for children highlights the salience of social support (or lack thereof) from partners for the pandemic stress process. Inequity can also create distress by generating feelings of unfairness (Greenstein 2009). This may be particularly relevant for understanding gender mental health gaps when we consider the accumulation of stress over the course of a multiyear pandemic rather than focusing on the first wave where an “all-hands-on-deck” mentality may have glossed over such concerns. As health risks have ebbed and flowed and responses to the pandemic shifted over time, so too will the salience of different stressors.

Mothers’ perception of more negative changes in work/care balance and career prospects given similar exposure to other work/care covariates was notable. This may reflect more qualitative differences in the experience of work and care not captured by our measures, such as working from home versus at a workplace. If, for example, mothers experienced greater pressure to combine caregiving with working from home than fathers did (e.g., Lyttelton, Zang, and Musick 2020), this could explain why they reported worse work/life balance, all else equal. Likewise, the salience of gender discrimination in the workplace may make mothers more concerned that working from home will trigger maternal bias and damage their careers.

Research has increasingly highlighted the importance of accounting for emotional and cognitive aspects of domestic and care work that are less visible and easily measured for understanding gendered experiences (e.g., Daminger 2019; Dean et al. 2022; Doucet 2023; Petts and Carlson 2023). The index of the mental load associated with managing relationships and family functioning was strongly related to mental health, and mothers’ greater exposure to this stressor was by far the largest work/care contributor to the gender gap. Findings thus reinforce the importance of attending to such dimensions of caregiving as a vital component of the stress process at the work/care interface. We expect that this is particularly relevant to later pandemic stages. With public health approaches shifting from broad measures to an emphasis on individual risk management, as well as growing polarization and misinformation about COVID-19, figuring out how best to keep family members safe, manage relationships, and foster children’s well-being has become more challenging.

Although mothers’ greater exposure to work/care stressors contributed to the overall gender mental health gap, it was notable that the relationship between stressors and mental health was similar for mothers and fathers overall. For example, both mothers and fathers were equally vulnerable to stressors associated with declining career prospects and with family functioning. Traditional work-family ideals that emphasize fathers’ responsibility for breadwinning and mothers’ for caregiving have eroded in recent decades in favor of dual earner-career ideals if not practices (Pedulla and Thébaud 2015). Men’s work-family conflict has likewise increased, with men and women now reporting similar levels (Nomaguchi 2009; Young and Schieman 2018). Our findings of similar vulnerability to work/care stressors are consistent with this trend, with both mothers’ and fathers’ mental health affected by threats to employment and family well-being.

At the same time, there were gender differences in the extent to which being in a family with high-risk members conditioned the pandemic stress process, reinforcing the importance of an intersectional analysis. All members of high-risk families had to deal with greater viral threats, but this situation typically contributed to greater exposure to work/care stressors only for mothers. Mothers in high-risk families reported a higher mental load associated with concerns about family functioning and greater concerns about balancing work/school and childcare relative to prepandemic baselines than other mothers. Mothers in high-risk families also experienced greater deterioration in mental health than other mothers when their relative share of caregiving increased. We did not find evidence of such divergence by high-risk status among fathers. Each of these differences contributed to the markedly worse mental health outcomes for mothers in high-risk families, who bore the largest pandemic mental health burden.

While our findings reveal that stressors magnifying mental health gaps between high-risk parents and others were greater among mothers, there is a similar pattern with respect to covariates that tended to attenuate differences. Circumstances that removed key resources but also reduced COVID-19 risks (such as reduced working hours and school closures) were typically associated with better mental health outcomes for parents in high-risk families relative to their non-high-risk counterparts. This general pattern was evident for both mothers and fathers, although the specific covariates varied. Throughout the pandemic, there has been an emphasis on “getting back to normal” for the sake of people’s mental health—our findings highlight that this perspective privileges those least at risk and whose mental health has been least affected. More broadly, our results demonstrate that high-risk health status, which overlaps with but is not reducible to disability as conventionally understood, is an important axis of inequality not only for physical health outcomes but also for mental health. Investments in targeted mental health supports and legal and organizational approaches to workplace flexibility that account for the heightened risk of not only employees but also their family members would be a helpful first step toward addressing these ongoing pandemic challenges and inequities.

Our analysis treated the household as a unit when considering high-risk status. This reflects the contagious nature of COVID-19 such that the actions of each member have ramifications for the risks of infecting the most vulnerable member(s). At the same time, the adaptations parents make and the relationship between work/care stressors and mental health may vary depending on who is at elevated risk. Parents may, for example, experience more stress about withdrawing children from school to protect a parent as opposed to a child. Other elements of social location also condition parents’ experiences of the pandemic, with implications for mental health gaps. Canadian mothers’ employment was more strongly affected among those with less education (Fuller and Qian 2021; Qian and Fuller 2020) and immigrants (Qin et al. 2022), while those deemed essential workers faced other challenges related to heightened viral risks and long work hours. Because we were interested in shifts in household divisions of labor, we restricted our analysis to partnered parents. But better mental health outcomes for high-risk families in the face of some employment and care disruptions may be conditional on partnered status. Weathering the loss of employment and care resources is no doubt easier for those with partners who can provide financial or caregiving support. Class status and financial security are also relevant here. We encourage more research to delve into these potential differences.

Our data were collected a year and a half into the pandemic, and the landscape has continued to evolve. Pandemic safety precautions have been dismantled, employers are requiring remote workers to return to the office, and most people who do not consider themselves at elevated risk appear to have resumed life more or less “as normal.” For such families, the pandemic impact on gender differences in mental health has probably receded. For high-risk families, this is unlikely to be the case. For some, the availability of vaccines for all age groups, Paxlovid, and the lower mortality associated with omicron-descended variants have reduced the threat of death from the acute, initial phase of infection compared with when our survey was conducted. Some families who identified as having a high-risk family member back in 2021 likely judge risks much lower today. However, for those who remain at substantial risk—severely immunocompromised people who derive limited protection from vaccines and for whom Paxlovid is often contraindicated—the situation has deteriorated in important ways. Removal of pandemic safety precautions has made avoiding infection increasingly difficult, especially for families with younger children who cannot effectively protect themselves with one-way masking. Unchecked transmission and viral mutation have also rendered ineffective the monoclonal antibodies that were first-line treatment for the immunocompromised.

Moreover, as the bulk of society “moves on,” prolonged social isolation and increasing stigmatization of those who continue to make visible efforts to avoid infection magnify threats to mental health. Parents face the dilemma of weighing infection risks against stoking children’s anxiety and inviting bullying and ostracization should their children continue to wear masks in schools and care settings where they are often the only one. This suggests that the mental load is becoming heavier for such families, increasing the mental health gap relative to those at lower risk. Devaluation of the lives and social inclusion of medically vulnerable people—apparent in the abandonment of measures to curb transmission and in the way that media and government officials frame COVID deaths^3^—is also a potent mental health stressor that deserves scrutiny. Government messaging and individual practices that focus on personal risk assessments irrespective of how actions can harm vulnerable others send the message that high-risk families do not matter, as does lack of employer recognition of the importance for some employees to be able to continue to work from home. Stigmatization, devaluation, and social exclusion can proliferate the stress of the pandemic for high-risk families and erode the social supports and relationships that might otherwise help buffer stressors associated with a heightened risk burden. As COVID-19 continues to spread, these issues, which we could not explicitly address in our study, are important avenues for both research and action.

## Supplemental Material

sj-docx-1-smh-10.1177_21568693231223549 – Supplemental material for The Work/Care Interface and Parents’ Mid-pandemic Mental Health: Inequalities at the Intersection of Gender and High-risk Household Status Supplemental material, sj-docx-1-smh-10.1177_21568693231223549 for The Work/Care Interface and Parents’ Mid-pandemic Mental Health: Inequalities at the Intersection of Gender and High-risk Household Status by Sylvia Fuller, Manlin Cai and Donna Lero in Society and Mental Health

## Notes

1. In Canada, emergency measures were introduced early in the pandemic to provide financial support for those who lost their job, but these typically did not fully replace lost earnings and ended in September 2020.

2. Remote work is another important aspect of employment status. Unfortunately, a survey error resulted in a large amount of missing data for the familydemic variable indexing actual remote work patterns. We tested expanding the employment status variable to account for access to remote work, which was not affected by this error, disaggregating the employed category into employed, no access to remote work, employed, access to remote work, and employed, remote work required. The expanded variable did not contribute to any of the mental health gaps we estimated nor did it result in substantive changes to other results.

3. When the deaths of medically vulnerable people from COVID-19 are acknowledged, their “comorbidities” are typically highlighted to downplay and normalize their deaths in both official announcements and media coverage.
