# Supplementary material for: The Work/Care Interface and Parents’ Mid-Pandemic Mental Health: Inequalities at the Intersection of Gender and High-Risk Household Status
Source: Soc Ment Health. 2024 Feb 14;15(1):75–93. doi: 10.1177/21568693231223549 (PMC11850158; doi:10.1177/21568693231223549)
Supplement: sj-docx-1-smh-10.1177_21568693231223549 – Supplemental material for The Work/Care Interface and Parents’ Mid-pandemic Mental Health: Inequalities at the Intersection of Gender and High-risk Household Status [file sj-docx-1-smh-10.1177_21568693231223549.docx]

ONLINE APPENDICES

Online Appendix A. Descriptive statistics by gender and high-risk family status (N = 2,782)

|  | Fathers (Mean/Prop.) | | | Mothers (Mean/Prop.) | | |
| --- | --- | --- | --- | --- | --- | --- |
|  | High-risk | Non-high-risk | Total | High-risk | Non-high-risk | Total |
| Current mental health | 2.78 | 2.90 | 2.87 | 2.42 | 2.67 | 2.60 |
| Care demands |  |  |  |  |  |  |
| Max. time of school/care closure | 1.60 | 1.52 | 1.54 | 1.73 | 1.59 | 1.63 |
| Max. time of school/care loss for quarantine/isolation |  |  |  |  |  |  |
| None | .58 | .64 | .63 | .54 | .64 | .61 |
| Less than a month | .29 | .23 | .24 | .35 | .25 | .27 |
| About a month or more | .14 | .13 | .13 | .12 | .12 | .12 |
| Max. time of school/care withdrawal |  |  |  |  |  |  |
| None | .66 | .71 | .70 | .63 | .72 | .70 |
| Less than a month | .10 | .11 | .11 | .09 | .07 | .08 |
| About a month | .03 | .03 | .03 | .04 | .05 | .05 |
| About 2-3 months | .05 | .04 | .05 | .07 | .05 | .05 |
| About 4-5 months | .03 | .02 | .02 | .03 | .02 | .02 |
| Six months or more | .13 | .08 | .09 | .14 | .09 | .10 |
| Changes in overall time spent on childcare |  |  |  |  |  |  |
| Much less time than before | .03 | .02 | .02 | .02 | .02 | .02 |
| Somewhat less time than before | .04 | .03 | .03 | .03 | .03 | .03 |
| About the same amount of time as before | .42 | .51 | .49 | .37 | .41 | .40 |
| Somewhat more time than before | .27 | .23 | .24 | .25 | .21 | .22 |
| Much more time than before | .23 | .21 | .21 | .33 | .32 | .32 |
| A greater share of childcare load than partner | .19 | .18 | .18 | .34 | .31 | .32 |
| Concerns about family functioning | 3.47 | 3.40 | 3.42 | 3.72 | 3.58 | 3.62 |
| Employment |  |  |  |  |  |  |
| Self-precipitated reduction in working hours | .06 | .05 | .05 | .12 | .09 | .10 |
| Employer-precipitated reduction in working hours | .06 | .09 | .09 | .06 | .06 | .06 |
| Leaving one's job for caregiving or health reasons | .01 | .00 | .01 | .04 | .02 | .02 |
| Losing a job | .06 | .06 | .06 | .06 | .06 | .06 |
| Being temporary laid off/furloughed | 1.09 | 1.08 | 1.08 | 1.08 | 1.07 | 1.07 |
| Current employment status |  |  |  |  |  |  |
| Employee | .92 | .93 | .93 | .78 | .80 | .80 |
| Self-employed | .02 | .02 | .02 | .02 | .01 | .01 |
| On leave | .03 | .02 | .03 | .11 | .11 | .11 |
| Unemployed | .02 | .02 | .02 | .04 | .03 | .03 |
| Out of labour force | .01 | .01 | .01 | .05 | .04 | .04 |
| Concerns about declining career prospects | 2.91 | 2.96 | 2.95 | 3.12 | 3.03 | 3.06 |
| Concerns about balancing work/school and childcare | 3.45 | 3.40 | 3.41 | 3.80 | 3.62 | 3.67 |
| Controls |  |  |  |  |  |  |
| Household income |  |  |  |  |  |  |
| Under $25,000 | .00 | .01 | .01 | .02 | .01 | .02 |
| $25,000 to less than $50,000 | .08 | .03 | .04 | .07 | .06 | .06 |
| $50,000 to less than $100,000 | .31 | .27 | .28 | .27 | .30 | .29 |
| $100,000 to less than $150,000 | .35 | .38 | .37 | .40 | .37 | .38 |
| $150,000 to less than $200,000 | .17 | .19 | .19 | .16 | .17 | .17 |
| Over $200,000 | .09 | .11 | .11 | .08 | .09 | .09 |
| Partner's current employment status |  |  |  |  |  |  |
| Employee | .61 | .63 | .63 | .82 | .83 | .83 |
| Self-employed | .07 | .08 | .08 | .06 | .09 | .08 |
| On leave | .13 | .11 | .11 | .03 | .03 | .03 |
| Unemployed | .04 | .03 | .03 | .04 | .02 | .03 |
| Out of labour force | .16 | .14 | .15 | .05 | .03 | .04 |
| Respondent with disability | .11 | .03 | .04 | .12 | .04 | .06 |
| Partner with disability | .10 | .02 | .04 | .09 | .02 | .04 |
| Child with disability | .10 | .03 | .05 | .10 | .04 | .06 |
| Pre-pandemic mental health | 3.33 | 3.47 | 3.43 | 3.18 | 3.28 | 3.26 |
| Age group |  |  |  |  |  |  |
| 20-34 | .23 | .28 | .27 | .30 | .41 | .38 |
| 35-44 | .57 | .58 | .58 | .62 | .52 | .55 |
| 45-59 | .19 | .14 | .15 | .09 | .07 | .07 |
| Age of youngest child |  |  |  |  |  |  |
| Under 1 year | .15 | .21 | .20 | .14 | .23 | .21 |
| Preschool | .60 | .58 | .59 | .59 | .56 | .57 |
| School-aged | .25 | .20 | .21 | .27 | .21 | .23 |

Online Appendix B1. Decomposition of gender gap

| Overall | | | | |
| --- | --- | --- | --- | --- |
| Father’s mental health | 2.884*** | | | |
| Mother’s mental health | 2.617*** | | | |
| Difference | 0.267*** | | | |
| Endowments | 0.229*** | | | |
| Coefficients | 0.015 | | | |
| Interaction | 0.023 | | | |
| Variables | Endowment contributions to the gap | Coefficient contributions to the gap | Coefficients for fathers | Coefficients for mothers |
| Max. time of school/care closure | 0.002 | -0.023 | -0.038 | -0.024 |
| Max. time of school/care loss for quarantine/isolation | 0.001 | 0.019 |  |  |
| None |  |  | 0.031 | -0.002 |
| Less than a month |  |  | 0.004 | -0.014 |
| About a month or more |  |  | -0.036 | 0.016 |
| Max. time of school/care withdrawal | -0.006* | 0.006 |  |  |
| None |  |  | -0.015 | -0.026 |
| Less than a month |  |  | 0.045 | -0.099 |
| About a month |  |  | -0.013 | 0.173 |
| About 2-3 months |  |  | -0.062 | -0.057 |
| About 4-5 months |  |  | 0.086 | -0.004 |
| Six months or more |  |  | -0.041 | 0.013 |
| Changes in overall time spent on childcare | 0.009 | 0.040 |  |  |
| Much less time than before |  |  | -0.141 | -0.146 |
| Somewhat less time than before |  |  | -0.210* | -0.080 |
| About the same amount of time as before |  |  | 0.209** | 0.101+ |
| Somewhat more time than before |  |  | 0.120* | 0.114 |
| Much more time than before |  |  | 0.022 | 0.011 |
| A greater share of childcare load than partner | 0.023** | 0.038 | -0.054 | -0.171*** |
| Concerns about family functioning | 0.100*** | -0.336 | -0.568*** | -0.476*** |
| Self-precipitated reduction in working hours | 0.001 | -0.001 | -0.023 | -0.013 |
| Employer-precipitated reduction in working hours | 0.004 | -0.015+ | -0.060 | 0.203 |
| Leaving one's job for caregiving or health reasons | 0.002 | 0.017 | 0.601 | -0.132 |
| Losing a job | -0.000 | 0.002 | 0.004 | -0.033 |
| Being temporary laid off/furloughed | 0.000 | 0.077 | 0.131 | 0.058 |
| Concerns about declining career prospects | 0.015+ | -0.137 | -0.192*** | -0.147*** |
| Concerns about balancing work/school and childcare | 0.013+ | 0.038 | -0.041 | -0.051+ |
| Household income | 0.002 | 0.003 |  |  |
| Under $25,000 |  |  | -0.087 | -0.252** |
| $25,000 to less than $50,000 |  |  | -0.054 | 0.112 |
| $50,000 to less than $100,000 |  |  | -0.129* | -0.023 |
| $100,000 to less than $150,000 |  |  | 0.068+ | -0.006 |
| $150,000 to less than $200,000 |  |  | 0.067 | 0.013 |
| Over $200,000 |  |  | 0.135*** | 0.156** |
| Respondent’s current employment status | 0.001 | -0.119 |  |  |
| Employee |  |  | -0.121 | 0.029 |
| Self-employed |  |  | -0.059 | -0.202 |
| On leave |  |  | -0.068 | -0.072 |
| Unemployed |  |  | -0.063 | 0.047 |
| Out of labour force |  |  | 0.310 | 0.198+ |
| Partner's current employment status | -0.023 | -0.118*** |  |  |
| Employee |  |  | -0.043 | 0.088* |
| Self-employed |  |  | -0.124 | 0.093 |
| On leave |  |  | 0.094* | 0.028 |
| Unemployed |  |  | 0.180 | -0.149 |
| Out of labour force |  |  | -0.107* | -0.059 |
| Respondent with disability | -0.001 | -0.013 | -0.172 | 0.058 |
| Partner with disability | -0.001 | -0.006 | -0.033 | 0.116 |
| Child with disability | 0.003 | 0.025* | 0.166 | -0.271* |
| Pre-pandemic mental health | 0.066*** | 0.298 | 0.496*** | 0.405*** |
| Age group | 0.018 | 0.036+ |  |  |
| 20-34 |  |  | -0.079** | -0.075 |
| 35-44 |  |  | 0.050+ | -0.033 |
| 45-59 |  |  | 0.029 | 0.108 |
| Age of youngest child | -0.001 | 0.022 |  |  |
| Under 1 year |  |  | -0.020 | 0.078*** |
| Preschool |  |  | -0.025 | -0.080*** |
| School-aged |  |  | 0.044 | 0.002 |

Note: *** p<.001, ** p<.01, * p<.05, + p<.1

Online Appendix B2. Decomposition of the gap between high-risk families and others, fathers

| Overall | | | | |
| --- | --- | --- | --- | --- |
| Non-high-risk family’s mental health | 2.901*** | | | |
| High-risk family’s mental health | 2.829*** | | | |
| Difference | 0.072 | | | |
| Endowments | -0.027 | | | |
| Coefficients | -0.072 | | | |
| Interaction | 0.172*** | | | |
| Variables | Endowment contributions to the gap | Coefficient contributions to the gap | Coefficients for non-high-risk family | Coefficients for high-risk family |
| Max. time of school/care closure | 0.002 | 0.120 | -0.013 | -0.09 |
| Max. time of school/care loss for quarantine/isolation | 0.010+ | -0.006 |  |  |
| None |  |  | 0.032 | 0.078 |
| Less than a month |  |  | 0.004 | -0.096 |
| About a month or more |  |  | -0.037 | 0.019 |
| Max. time of school/care withdrawal | -0.018 | 0.216*** |  |  |
| None |  |  | 0.033 | -0.248*** |
| Less than a month |  |  | 0.192** | -0.411* |
| About a month |  |  | -0.156 | 0.426** |
| About 2-3 months |  |  | 0.003 | -0.134 |
| About 4-5 months |  |  | -0.016 | 0.354+ |
| Six months or more |  |  | -0.055 | 0.014 |
| Changes in overall time spent on childcare | 0.017 | 0.161*** |  |  |
| Much less time than before |  |  | -0.377*** | 0.355 |
| Somewhat less time than before |  |  | -0.186 | -0.189 |
| About the same amount of time as before |  |  | 0.234*** | 0.294** |
| Somewhat more time than before |  |  | 0.228*** | -0.117 |
| Much more time than before |  |  | 0.101+ | -0.344*** |
| A greater share of childcare load than partner | 0.000 | -0.037 | -0.093 | 0.099 |
| Concerns about family functioning | 0.022 | 0.021 | -0.541*** | -0.547*** |
| Self-precipitated reduction in working hours | -0.001 | -0.012 | -0.126 | 0.030 |
| Employer-precipitated reduction in working hours | -0.003 | 0.008 | -0.025 | -0.149 |
| Leaving one's job for caregiving or health reasons | -0.013 | -0.022 | -0.277 | 1.433** |
| Losing a job | -0.002 | 0.011 | 0.071 | -0.143 |
| Being temporary laid off/furloughed | -0.002 | -0.109 | 0.089 | 0.191 |
| Concerns about declining career prospects | -0.020 | 0.316 | -0.169** | -0.278*** |
| Concerns about balancing work/school and childcare | 0.002 | 0.599 | -0.010 | -0.183 |
| Household income | 0.001 | -0.227 |  |  |
| Under $25,000 |  |  | 0.043 | -1.158 |
| $25,000 to less than $50,000 |  |  | -0.100 | 0.151 |
| $50,000 to less than $100,000 |  |  | -0.152+ | 0.111 |
| $100,000 to less than $150,000 |  |  | 0.063 | 0.235* |
| $150,000 to less than $200,000 |  |  | 0.055 | 0.340** |
| Over $200,000 |  |  | 0.09+ | 0.322+ |
| Respondent’s current employment status | 0.000 | 0.436 |  |  |
| Employee |  |  | 0.018 | -0.429 |
| Self-employed |  |  | 0.114 | 0.085 |
| On leave |  |  | 0.303 | -1.034** |
| Unemployed |  |  | -0.123 | 0.478 |
| Out of labour force |  |  | -0.312** | 0.900 |
| Partner's current employment status | -0.006 | -0.073 |  |  |
| Employee |  |  | -0.052 | 0.018 |
| Self-employed |  |  | -0.104 | -0.156 |
| On leave |  |  | 0.072 | 0.201** |
| Unemployed |  |  | 0.246 | -0.101 |
| Out of labour force |  |  | -0.162* | 0.038 |
| Respondent with disability | -0.001 | -0.029 | -0.277 | 0.019 |
| Partner with disability | -0.016* | -0.063* | -0.455+ | 0.208** |
| Child with disability | -0.009 | -0.003 | 0.107 | 0.134 |
| Pre-pandemic mental health | 0.037+ | 0.523** | 0.545*** | 0.389*** |
| Age group | -0.006 | 0.002 |  |  |
| 20-34 |  |  | -0.063 | -0.094 |
| 35-44 |  |  | 0.049 | 0.041 |
| 45-59 |  |  | 0.014 | 0.053 |
| Age of youngest child | -0.021* | -0.041 |  |  |
| Under 1 year |  |  | 0.033 | -0.213*** |
| Preschool |  |  | -0.031 | 0.023 |
| School-aged |  |  | -0.003 | 0.190** |

Note: *** p<.001, ** p<.01, * p<.05, + p<.1

Online Appendix B3. Decomposition of the gap between high-risk families and others, mothers

| Overall | | | | |
| --- | --- | --- | --- | --- |
| Non-high-risk family’s mental health | 2.687*** | | | |
| High-risk family’s mental health | 2.443*** | | | |
| Difference | 0.243*** | | | |
| Endowments | 0.138*** | | | |
| Coefficients | 0.095 | | | |
| Interaction | 0.011 | | | |
| Variables | Endowment contributions to the gap | Coefficient contributions to the gap | Coefficients for non-high-risk family | Coefficients for high-risk family |
| Max. time of school/care closure | -0.007 | -0.220*** | -0.087*** | 0.027 |
| Max. time of school/care loss for quarantine/isolation | 0.000 | 0.063* |  |  |
| None |  |  | 0.036 | -0.075+ |
| Less than a month |  |  | -0.012 | -0.079* |
| About a month or more |  |  | -0.024 | 0.154** |
| Max. time of school/care withdrawal | 0.003 | 0.045 |  |  |
| None |  |  | -0.035 | -0.062 |
| Less than a month |  |  | -0.069 | -0.152 |
| About a month |  |  | 0.065 | 0.308 |
| About 2-3 months |  |  | -0.177 | 0.133 |
| About 4-5 months |  |  | 0.021 | -0.086 |
| Six months or more |  |  | 0.196 | -0.141 |
| Changes in overall time spent on childcare | -0.013 | -0.154 |  |  |
| Much less time than before |  |  | -0.196 | -0.01 |
| Somewhat less time than before |  |  | 0.094 | -0.623* |
| About the same amount of time as before |  |  | 0.104+ | 0.169+ |
| Somewhat more time than before |  |  | 0.081 | 0.242*** |
| Much more time than before |  |  | -0.084 | 0.222 |
| A greater share of childcare load than partner | 0.015 | 0.103* | -0.076 | -0.365*** |
| Concerns about family functioning | 0.065** | -0.339 | -0.508*** | -0.417** |
| Self-precipitated reduction in working hours | -0.003 | -0.038 | -0.139* | 0.205 |
| Employer-precipitated reduction in working hours | -0.002 | -0.011 | 0.13 | 0.301* |
| Leaving one's job for caregiving or health reasons | 0.007 | 0.010 | -0.014 | -0.25 |
| Losing a job | 0.000 | 0.002 | -0.033 | -0.068 |
| Being temporary laid off/furloughed | -0.001 | 0.731** | 0.271* | -0.42** |
| Concerns about declining career prospects | 0.012 | -0.134 | -0.155*** | -0.112** |
| Concerns about balancing work/school and childcare | 0.022* | 0.279 | -0.011 | -0.083** |
| Household income | -0.002 | -0.098 |  |  |
| Under $25,000 |  |  | -0.042 | -0.685* |
| $25,000 to less than $50,000 |  |  | -0.033 | 0.382** |
| $50,000 to less than $100,000 |  |  | -0.064 | 0.026 |
| $100,000 to less than $150,000 |  |  | -0.031 | 0.016 |
| $150,000 to less than $200,000 |  |  | -0.062+ | 0.263 |
| Over $200,000 |  |  | 0.233*** | -0.002 |
| Respondent’s current employment status | -0.007 | 0.096 |  |  |
| Employee |  |  | 0.046 | -0.058 |
| Self-employed |  |  | -0.118 | -0.097 |
| On leave |  |  | -0.006 | -0.239** |
| Unemployed |  |  | 0.015 | 0.118 |
| Out of labour force |  |  | 0.063 | 0.276 |
| Partner's current employment status | 0.010 | -0.049 |  |  |
| Employee |  |  | 0.068 | 0.13* |
| Self-employed |  |  | 0.026 | 0.172 |
| On leave |  |  | 0.185+ | -0.12 |
| Unemployed |  |  | -0.286 | 0.089 |
| Out of labour force |  |  | 0.008 | -0.271 |
| Respondent with disability | 0.004 | 0.030 | 0.205* | -0.058 |
| Partner with disability | -0.005 | 0.003 | 0.113 | 0.08 |
| Child with disability | 0.007 | -0.024* | -0.319** | -0.099 |
| Pre-pandemic mental health | 0.021* | 0.455*** | 0.44*** | 0.299*** |
| Age group | -0.007 | -0.003 |  |  |
| 20-34 |  |  | -0.069 | -0.043 |
| 35-44 |  |  | -0.022 | -0.022 |
| 45-59 |  |  | 0.091 | 0.065 |
| Age of youngest child | 0.017 | 0.006 |  |  |
| Under 1 year |  |  | 0.055 | 0.103 |
| Preschool |  |  | -0.08* | -0.068 |
| School-aged |  |  | 0.025 | -0.035 |

Note: *** p<.001, ** p<.01, * p<.05, + p<.1
